# Cognitive behavioral therapy to reduce persistent postsurgical pain following internal fixation of extremity fractures (COPE): Rationale for a randomized controlled trial

**DOI:** 10.1080/24740527.2019.1615370

**Published:** 2019-07-30

**Authors:** Matilda E. Nowakowski, Randi E. McCabe, Jason W. Busse

**Affiliations:** a Department of Psychiatry and Behavioural Neurosciences, McMaster University, Hamilton, Ontario, Canada; b Chronic Pain Clinic, St. Joseph’s Healthcare Hamilton, Hamilton, Ontario, Canada; c Anxiety Treatment and Research Clinic, St. Joseph’s Healthcare Hamilton, Hamilton, Ontario, Canada; d The Michael G. DeGroote Institute for Pain Research and Care, McMaster University, Hamilton, Ontario, Canada; e Department of Anesthesia, McMaster University, Hamilton, Ontario, Canada; f Department of Health Research Methods, Evidence, and Impact, McMaster University, Hamilton, Ontario, Canada; g The Michael G. DeGroote Centre for Medicinal Cannabis Research, McMaster University, Hamilton, Ontario, Canada

**Keywords:** persistent postsurgical pain, cognitive behavioral therapy, extremity fractures

## Abstract

**Background**: Approximately half of all patients who undergo surgical repair of extremity fractures report persistent postsurgical pain (PPSP) at 1-year post-surgery. Psychological factors such as anxiety, depression, catastrophization, poor coping, high somatic complaints, and pessimism about recovery are risk factors for the development of PPSP. It is possible that interventions such as cognitive behavior therapy (CBT) that target psychological factors may reduce the incidence of PPSP in this population.

**Aims**: The current report reviews the role of psychological factors in the development of PPSP and discusses the rationale and protocol development for a multi-site randomized-controlled trial investigating the effectiveness of CBT in reducing PPSP in patients with surgically treated extremity fractures.

## RÉSUMÉ

**Contexte**: Près de la moitié des patients qui sont opérés pour des fractures aux extrémités font état de douleur postopératoire persistante un an après la chirurgie. Des facteurs psychologiques tels que l’anxiété, la dépression, la catastrophisation, une piètre capacité d’adaptation, une somatisation élevée et le pessimisme quant à la récupération sont des facteurs de risque pour le développement de la douleur postopératoire persistante. Il est possible que des interventions ciblant les facteurs psychologiques, telles que la thérapie cognitivo-comportementale (TCC), puissent réduire l’incidence de la douleur postopératoire persistante dans cette population.

**Objectifs**: Le présent rapport examine le rôle des facteurs psychologiques dans le développement de la douleur postopératoire persistante et discute de la justification et du développement d’un protocole pour un essai contrôlé randomisé multisite portant sur l’efficacité de la TCC pour réduire la douleur postopératoire persistante chez les patients ayant été opérés pour des fractures aux extrémités.

Persistent noncancer pain affects one in five Canadians^1^ and can have a major impact on individuals’ quality of life, including ability to return to work and partake in daily activities.^2^ Chronic pain is also associated with significant direct and indirect health care costs, estimated at between $43 and $60 billion per year in Canada.^3,4^ Surgery and trauma are common triggering events for the development of chronic pain.^5^ A UK survey of over 5000 patients found that 41.2% attributed their chronic pain to a traumatic event (18.7%) or surgery (22.5%), with 60% of such patients experiencing chronic pain for over 2 years after the trauma or surgery and 75% rating their pain as moderate or severe.^6^

Although acute pain is normal following surgery, the past 2 decades has seen an increased recognition that the incidence of persistent postsurgical pain (PPSP) is higher than previously recognized and is a significant contributor to the burden of chronic pain.^7^ Persistent postsurgical pain is defined by four criteria: (1) pain onset following surgery or tissue trauma, (2) pain is in an area preceding surgery or tissue trauma, (3) pain persists for at least 3 months following surgery, and (4) pain is not better explained by other factors such as infection, malignancy, a pre-existing pain condition, or any other alternative cause.^8^ It is estimated that between 10% and 85% of patients undergoing surgery will experience PPSP, and 2% to 10% of these patients develop severe PPSP.^9–12^ The incidence of PPSP varies according to type of surgery, with limb amputation, cardiac, breast, and orthopedic surgeries associated with higher rates.^7^

## Extremity fractures and persistent postsurgical pain

Clinical outcomes following operatively managed fractures of the extremities are variable, and many patients report PPSP and disability 1 year after surgery and beyond.^13–15^ In a recent trial involving patients with open extremity fractures, 65% of patients endorsed moderate to very severe pain and 35% endorsed moderate to extreme pain interference at 1 year after surgery.^13^ A systematic review of 20 observational studies of traumatic tibial fracture repairs found that 47.4% (range 10%–86%) of patients experienced PPSP at an average of 23.9 months after surgery.^16^

## Psychological factors in persistent postsurgical pain

The transition from acute pain to PPSP is a complex process that is influenced by a variety of risk factors in the preoperative, intraoperative, and postoperative periods, including demographic factors, genetics, psychological factors, pain levels (preoperative and acute postoperative), type of surgery, opioid use, and comorbidities.^7,17^ Although many of these factors are nonmodifiable (e.g., age, gender, genetics), a number of predictors, including psychological factors, are potentially modifiable during the perioperative period.

The relationship between psychological factors and pain is well established. Anxiety, depression, pain catastrophizing (i.e., exaggerated negative cognitions about pain; for review, see Leung^18^), fear avoidance behaviors (e.g., avoiding activities for fear of pain), and poor coping strategies have all been shown to be positively related with chronic pain.^19^ More recently, a variety of psychological factors have also been associated with increased risk for the development of PPSP, including anxiety,^20–23^ depression,^24^ and pain catastrophizing,^23,25–27^ suggesting that patients’ baseline psychological well-being as well as cognitions, emotions, and behavioral reactions to surgery and the experience of pain are associated with prognosis.

Busse et al.^28^ recently developed the Somatic Pre-Occupation and Coping (SPOC) Questionnaire to identify unhelpful illness beliefs that are predictive of poorer outcomes (pain and function) postfracture. This 27-item questionnaire assesses four domains: somatic complaints (e.g., “How often have you experienced stiff joints in the past week?”), coping (e.g., “There is a lot I can do to control my injury-related symptoms”), energy (“Have you been feeling low in energy and slowed down during the past week?”), and optimism (“Have you felt that you could not overcome your difficulties during the past week?”). Higher scores on the SPOC Questionnaire are indicative of high somatic complaints, low energy, poor coping, and pessimism about recovery.^28^ Scores on the SPOC questionnaire at 6 weeks postsurgery have been shown to be more strongly predictive of physical and emotional functioning, unemployment, and quality of life at 1 year postsurgery than other known risk factors for the development of PPSP such as gender, site and severity of the fracture, smoking status, type of fixation, and whether or not the injury was work related.^28–30^

In a study of 267 patients undergoing traumatic tibial fracture repair, the rates of PPSP at 1 year postsurgery were 37.6% for patients with low SPOC scores at 6 weeks postsurgery, 54.1% for patients with intermediate SPOC scores at 6 weeks postsurgery, and 81.6% for patients with high SPOC scores at 6 weeks postsurgery.^30^ Patients who had high SPOC scores at 6 weeks postsurgery were seven times more likely to report PPSP and ten times more likely to endorse pain interference at 1 year postsurgery compared to patients with low SPOC scores at 6 weeks post tibial fixation.^30^ In another study of 1560 patients undergoing surgical fixation for an extremity fracture, patients with high SPOC scores at 6 weeks postsurgery were six times more likely to report persistent pain and at least moderate levels of pain interference at 1 year postsurgery compared to patients with low SPOC scores.^29^ As such, the beliefs that patients hold about their pain and recovery process appear to have a powerful effect on outcome and suggest the possibility that patients with fractures who exhibit unhelpful illness beliefs can be identified and targeted for concurrent therapy designed to modify such cognitions and improve prognosis.

Cognitive behavioral therapy (CBT) is a first-line psychological treatment for patients with chronic pain that focuses on the interrelationship between cognitions, emotions, physical sensations, and behaviors to understand and address patients’ current difficulties with a focus on helping patients learn new skills and strategies to cope with their chronic pain and to modify maladaptive cognitive and behavioral responses to chronic pain.^31,32^ Most CBT protocols include some combination of psychoeducation, goal-setting, relaxation strategies, cognitive restructuring (i.e., identifying and modifying unhelpful cognitions), problem solving, time-based pacing, communication strategies, and relapse prevention. More recently, there has been increasing interest in the utilization of CBT during the perioperative period as a strategy to decrease the risk of developing PPSP. A recent meta-analysis of 15 randomized controlled trials investigating the effectiveness of perioperative psychotherapy for patients undergoing a variety of surgical interventions found that patients who received active psychological interventions (CBT, relaxation strategies, or both) had significantly less persistent pain and physical impairment at 3- to 30-month follow-up compared to patients who received treatment as usual.^33^ Notably, just receiving education about coping strategies with no active psychological intervention did not affect the severity of persistent pain or physical impairment at follow-up (test of interaction *P* = 0.01 for both outcomes).

Looking specifically at the effects of CBT and relaxation strategies for reducing PPSP following orthopedic surgeries, studies to date have solely focused on hip and knee replacements.^33^ No studies have examined the effects of CBT in the reduction of PPSP in surgically managed acute closed or open extremity fractures. This is an important area for investigation because acute closed or open extremity fractures differ from joint replacement in a number of ways, including the sudden onset of the fracture and necessity for immediate surgical management, high rate of traumatic mechanisms of injury (e.g., motor vehicle accident, crush injury, direct trauma), and often inexperience with ill health due to the relatively young mean age of occurrence.^30^

## The proposed study

The **Co**gnitive Behavioural Therapy to Reduce **P**ersistent Post-Surgical Pain Following Internal Fixation of **E**xtremity Fractures (COPE) study was developed to examine the efficacy of CBT in the reduction of PPSP and disability in patients undergoing fixation of extremity fractures. This is a multisite randomized controlled trial that will recruit 1000 adult patients undergoing fixation surgery for an acute closed or open extremity fracture across approximately 15 clinical sites in Canada and, potentially, The Netherlands.

Inclusion criteria will be as follows: (1) males and females aged 18 years or older; (2) acute open or closed fracture of the appendicular skeleton; (3) fracture treated operatively with internal fixation; (4) screened for eligibility within 6 weeks of fracture; (5) cognitive ability and language skills to participate in CBT; and (6) able to start CBT within 8 weeks of fracture. Exclusion criteria will include (1) fragility fracture, (2) stress fracture, (3) concomitant injury that is likely to impair functioning for as long as or longer than the extremity fracture, (4) active psychosis, (5) active suicidality, (6) active substance use disorder, (7) already participating in or planning to start a psychological treatment within the duration of the study (i.e., 12 months), (8) anticipated problems with partaking in the CBT sessions or completing follow-up appointments (e.g., lack of time), and (9) incarceration.

Patients will be randomized to one of two treatment arms: (1) CBT or (2) care as usual. Randomization will be stratified by clinical site, sex, fracture type (open or closed), and illness beliefs (SPOC score ≥ 48 versus SPOC score < 48). Patients randomized to psychotherapy will be able to select between six sessions of telephone CBT (1-h sessions) or 6 weeks of online CBT with asynchronous communication with a therapist. Both CBT interventions will follow the same treatment protocol. The rationale for including a choice of online or telephone CBT was guided by preliminary challenges in recruiting participants for face-to-face CBT due to scheduling and transportation difficulties and evidence that online or telephone-administered CBT is equally effective to in-person CBT^34^ and that online CBT is effective for chronic pain.^35^ As such, allowing participants the option of selecting between online and telephone CBT will increase the accessibility and feasibility of the study. Patients will complete follow-up assessments of pain intensity, pain interference, and physical and emotional functioning at baseline (4–8 weeks postfracture) and 3, 6, 9, and 12 months postsurgery. The statistical analysis plan is detailed in the Appendix.

The CBT protocol is outlined below, including the rationale for inclusion of different components of the protocol. Although the current protocol includes a number of components that are included in standard chronic pain protocols, the presentation of the components in the current study differs because the focus is on individuals at risk for development of chronic pain. Thus, the emphasis is on prevention and addressing vulnerability factors, including beliefs about pain and fear of movement, that may play a role in the development of PPSP.

### Week 1

Week 1 will provide an introduction to the principles of CBT (module 1), pain education (module 2), relaxation strategies (module 3), and goal-setting (module 4). Education about the nature of pain will discuss differences between acute and chronic pain, psychological factors that contribute to the transition from acute to chronic pain, the gate control theory of pain, and the role of central sensitization in chronic pain. The goal of pain education is to provide a better understanding of the complex nature of pain and a rationale for the different strategies that will be taught throughout the treatment program, including the importance of self-management strategies in the recovery process. Research evidence in chronic musculoskeletal pain has shown that pain neuroscience education reduces pain intensity, improves functioning, decreases disability, and decreases pain catastrophizing.^36^ Relaxation strategies (diaphragmatic breathing) will be presented as a tool to manage pain flares and stress related to the recovery process. Participant values and goal-setting using the SMART goal formula (specific, measurable, attainable, relevant, and time-bound) will be included to identify values-based actions that the participant can partake in postinjury to build hopefulness rather than focusing on losses and what the participant is unable to do postinjury.

### Week 2

Discussion of the CBT model and the interconnectedness of thoughts, emotions, behaviors, and physical sensations in the experience of pain (module 5) and introduction to cognitive restructuring (module 6) will occur in week 2. Cognitive restructuring is a CBT strategy that is aimed at identifying negative automatic thoughts and relevant cognitive distortions (i.e., unhelpful thinking patterns) and learning strategies to generate more balanced/helpful thoughts. The aim of cognitive restructuring is to teach a strategy to address unhelpful illness beliefs that are associated with the development of PPSP.^28–30^ Participants’ responses on the four domains of the SPOC questionnaire will guide the cognitive restructuring, particularly identifying and modifying unhelpful beliefs related to the pain experience and ability to cope with the recovery process.

### Week 3

Week 3 will provide an introduction to the fear avoidance model and modification of behavioral responses to pain (module 7). The fear avoidance model states that recovery following an injury follows one of two pathways depending on the interpretation of pain. If pain during the recovery process is interpreted as a nonthreatening normal part of recovery, then there is a gradual return to normal activities. On the other hand, if pain during the recovery process is interpreted as threatening and dangerous, then anxiety and fear develop, leading to avoidance of activities and disuse of the affected limb and a prolonged pain experience.^37^ As such, week 3 will focus on identification of any avoided and/or feared movements or activities that have been deemed as safe and engagement in gradual exposures to the feared/avoided movements or activities, noting anxious predictions, actual outcomes, and learning. The goal of the exposures is for participants to acquire accurate data as to the “danger” of activities or movements and to learn that if they do experience pain with the activity or movement, they are able to cope with it. Therapists will be in contact with other members of the participants’ health care team (e.g., surgeons, physiotherapists) as needed during this week.

### Week 4

An introduction to time-based pacing (module 8) and managing pain flares (module 9 and 10) will occur in week 4. Time-based pacing, a self-management strategy that is commonly used in chronic pain to address under- and overactivity, focuses on using behaviors such as breaks and switching tasks in a quota-contingent manner (e.g., take a break after doing the dishes for 10 min) rather than in a pain-contingent manner (e.g., take a break from the dishes when the pain increases to an 8 out of 10) in order to increase functioning despite the pain.^38^ Time-based pacing will be introduced as another strategy to help with engagement in feared activities/movements and accomplishing SMART goals. The goal of time-based pacing is to provide a starting point for engagement in activities and to gradually increase activity time as strength and stamina increase. The discussion about managing pain flares will include a normalization of the experience of pain flares throughout the recovery process and discussion of strategies for managing pain flares (e.g., diaphragmatic breathing, cognitive restructuring, engagement in a pleasurable activity, distraction, time-based pacing of activities, asking for help if needed, trying to maintain a regular schedule as much as possible despite the pain flare).

### Week 5

Week 5 will provide an introduction to mindfulness and acceptance (module 11), pain medications (module 12), and managing setbacks (module 13). When recovering from surgery, there is often a tendency to judge pain and progress negatively, which can lead to further negative emotions and a worsening of the pain experience. The concepts of mindfulness and acceptance and practicing of the body scan aim to help participants accept the present moment and to place more energy and focus onto what they can control in their recovery process rather than placing time and energy into negative emotions and what they cannot control. Week 5 will also include a discussion about use of pain medications and, if appropriate, will help participants prepare for any necessary discussions with their family physician around their use of pain medications.

### Week 6

Summarizing and preparing for the future (module 14) will take place in week 6. Participants will have an opportunity to reflect on the strategies and changes they have made throughout the 6-week program and to identify next steps with regards to maintaining and progressing forward on gains.

### Optional materials

Therapists will have access to optional modules that include problem solving, sleep hygiene, and further relaxation strategies (autogenic training, visual imagery, and mini-relaxation) that they can use as needed with patients (e.g., if there are significant sleep difficulties that are negatively impacting the recovery process, if patients are not finding the diaphragmatic breathing helpful as a relaxation strategy, if patients are dealing with a significant psychosocial stressor that is impeding their recovery).

## Clinical implications

Given the growing evidence that patients’ beliefs and emotional functioning play an important role in the development of persistent pain after fracture repair, research that investigates potential interventions for the reduction of PPSP is urgently needed, especially in high-risk populations. The proposed study will be the largest and only multisite study to date on the efficacy of CBT in the prevention of PPSP after extremity fracture repair. The results of this study may have significant implications for the implementation of interventions targeting beliefs and coping strategies to reduce the onset of persistent pain in patients with surgically managed extremity fractures.

## Appendix: Statistical analysis plan

The analysis and reporting of results will follow the CONSORT guidelines for reporting of randomized controlled trials. The process of participant enrollment and flow throughout the study will be summarized using a flow diagram. Participant demographics, fracture characteristics, fracture management details, and compliance with CBT will be summarized by treatment group using descriptive summary measures, expressed as mean (standard deviation) or median (interquartile range) for continuous variables depending on the distribution and number (percentage) for categorical variables.

An intention-to-treat principle will be used to analyze all primary and secondary outcomes. The date on which the participant’s fracture(s) occurred will be used as the starting point for all time-to-event analyses. Assuming that the data will be missing at random, multiple imputation will be used to address any missing data.^39^ We will conduct a sensitivity analysis using nonlinear multiple imputation to deal with missing observations.^40,41^All statistical tests will be performed using two-sided tests at the 0.05 level of significance. For all models, the results will be expressed as effect (odds ratios for binary outcomes and mean difference for continuous outcomes), corresponding two-sided 95% confidence intervals, and associated *P* values. *P* values will be reported to three decimal places with values less than 0.001 reported as <0.001. All analyses will be performed using SAS.

### Primary analysis

We will compare the proportion of participants who meet the criteria for moderate to severe PPSP in the CBT treatment group with the proportion of participants who meet the criteria for moderate to severe PPSP in the usual care group, over 12 months postfracture, using logistic regression. The primary analyses will be adjusted by clinical site, sex, any open fracture versus no open fracture, and greater illness beliefs versus lesser illness beliefs (based on SPOC score; Table A1). We will combine sites that enroll fewer than ten participants to give more power to a test for differences between sites, as long as this results in aggregating no more than 25% of all enrolled participants.

### Secondary analyses

*Physical and mental functioning over time*: We will score the Short Form-36 (SF-36) as per the developers’ guidelines to obtain the Physical Component Score (PCS) and Mental Component Score (MCS) score for each participant. We will compare the mean PCS and MCS scores over time, adjusting for the baseline PCS and MCS scores, between the CBT group and the usual care group using longitudinal analysis (Table A2). The analyses will also be adjusted by clinical site, sex, any open fracture versus no open fracture, and greater illness beliefs versus lesser illness beliefs (based on SPOC score).

*Return to function*: We will use logistic regression to determine whether the proportion of participants who report a return to ≥80% of their pre-injury function over 12 months is greater in the CBT group compared to the usual care group (Table A2). The proportion of participants who have returned to full function with respect to work, leisure activities, and activities around the home will also be compared between the CBT and the usual care groups over 12 months postfracture using logistic regression. The analyses will be adjusted for clinical site, sex, any open fracture versus no open fracture, and greater illness beliefs versus lesser illness beliefs (based on SPOC score).

*Pain over time*: We will score the Brief Pain Inventory (Short Form) (BPI-SF) as per the developers’ guidelines and obtain the average pain severity score over time and the pain interference score over time. We will use longitudinal analyses, adjusting for baseline scores, to determine whether mean pain severity scores and mean pain interference scores over time are lower in the CBT treatment group compared to the usual care treatment group (Table A2). The analyses will be adjusted for clinical site, sex, any open fracture versus no open fracture, and greater illness beliefs versus lesser illness beliefs (based on SPOC score).

*Opioid use over time*: Logistic regression models will be used to explore differences between the treatment groups in the proportion of participants prescribed opioids at 6 and 12 months postfracture. The analyses will also be adjusted by clinical site, sex, any open fracture versus no open fracture, and greater illness beliefs versus lesser illness beliefs (based on SPOC score).

### Subgroup analysis

Three subgroup analyses will be performed for both primary outcome measures: (1) male versus female; (2) any open fracture versus no open fracture; and (3) greater illness beliefs (defined as SPOC score ≥48) versus lesser illness beliefs (SPOC score <48). These analyses will be performed by comparing the effect estimates in both groups and calculating a test of interaction between the subgroup variable and the treatment group variable. We hypothesize that effect will differ by subgroup, with larger effects being seen in female participants,^42^ participants with open fractures,^43^ and participants with greater illness beliefs (Table A3). These analyses will be exploratory in nature and will be approached and reported in accordance with best practices and guidelines for subgroup analyses.^44^

### Sensitivity analysis

The following sensitivity analyses will be performed to explore the robustness of our findings: (1) Different correlation structures for the error: Although the generalized estimating equation method is robust to misclassification of correlation structure, we will re-examine the generalized estimating equation analysis assuming an unstructured error structure to allow for unequal number of participants within different clusters and periods and (2) complete case analysis.^41^

### Economic analysis

At each follow-up visit, participants employed at study start will be asked whether they have returned to work without limitations, returned to work with limited duties, or not returned to work. Participants will also be asked whether they have received physiotherapy or occupational therapy or have had any secondary procedures related to their fracture(s). The SF-36 allows the calculation of a health utility score, which is necessary to calculate quality-adjusted life years (QALYs). QALYs combine quantity of life with health-related quality of life and are used in cost-effectiveness analyses to compare outcomes between interventions. We will calculate QALYs for each intervention by weighing the utility scores by time spent in health states using an area under the curve approach. We will evaluate statistical differences in QALYs between CBT and usual care using a generalized linear model with log link and gamma distribution.

If neither strategy is found to be dominant (i.e., less costly and better outcomes), we will calculate the incremental cost-effectiveness ratios by calculating the difference in cost between CBT and care as usual divided by the difference in their effect (i.e., QALYs). Uncertainty regarding costs and QALYs due to sampling variability associated with the trial will be measured using nonparametric bootstrapping techniques. Cost-effectiveness acceptability curves will be used to present the probability of CBT to be cost-effective at two commonly cited willingness-to-pay thresholds ($50 000/QALY gained; $100 000/QALY gained).^45^ Analyses will be conducted from payer (direct costs) and societal (direct and indirect costs) perspectives over a 1-year time horizon. Indirect costs will be estimated by the value of wage loss incurred for those employed at the time of study.

### Interim analysis

No interim analysis will be conducted to avoid spuriously inflated estimates of treatment effect,^46,47^ and the trial will not be stopped early for benefit.

**Table A1.** Primary analysis overview.

Objective	Outcome		Hypothesis	Method of analysis
	Name	Type		
To determine whether CBT reduces the prevalence of moderate to severe PPSP over 12 months postfracture.	PPSP as defined by the WHO, and of ≥4/10 severity	Binary	The prevalence of PPSP over 12 months postfracture will be lower in the CBT treatment group compared to the usual care group	Logistic regression, adjusting for clinical site, sex, any open fracture versus no open fracture, and greater versus lesser illness beliefs

CBT = cognitive behavioral therapy; PPSP = persistent postsurgical pain; WHO = World Health Organization.

**Table A2.** Secondary analyses.

Objective	Outcome		Hypothesis	Method of analysis
	Name	Type		
Secondary objective 1				
To determine whether CBT improves physical and mental functioning over 12 months postfracture	SF-36 PCS	Continuous	Participants receiving CBT will have higher SF-36 PCS scores over 12 months compared to participants who do not receive CBT	Longitudinal analysis, adjusting for baseline SF-36 PCS
	SF-36 MCS	Continuous	Participants receiving CBT will have higher SF-36 MCS scores over 12 months compared to participants who do not receive CBT	Longitudinal analysis, adjusting for baseline SF-36 MCS
Secondary objective 2				
To determine whether CBT improves return to function over 12 months postfracture	Return to function score of 80%	Binary	The proportion of participants who report ≥80% of pre-injury functioning will be greater in the CBT group than the usual care group over 12 months postfracture	Logistic regression
	Return to full function with respect to work, leisure activities, and responsibilities around the home	Binary	The proportion of participants who have returned, without limitations, to work, leisure activities, and responsibilities around the home will be higher in participants in the CBT group than in the usual care group	Logistic regression
Secondary objective 3				
To determine whether CBT reduces pain over 12 months postfracture	BPI-SF average pain severity score	Continuous	Participants receiving CBT will have lower pain severity scores over 12 months compared to participants who do not receive CBT	Longitudinal analysis, adjusting for baseline scores
	BPI-SF pain interference score	Continuous	Participants receiving CBT will have lower pain interference scores over 12 months compared to participants who do not receive CBT	Longitudinal analysis, adjusting for baseline scores
Secondary objective 4				
To determine whether CBT reduces the proportion of participants prescribed opioid class medications at 6 months and 12 months	Taking an opioid class medication	Binary	The proportion of participants prescribed opioids at 6 months and 12 months will be lower in participants receiving the CBT compared to participants who do not receive CBT	Logistic regression

CBT = cognitive behavioral therapy; SF-36 = Short Form-36; PCS = Physical Component Score; MCS = Mental Component Score; BPI-SF = Brief Pain Inventory (Short Form).

**Table A3.** Subgroup analyses.

Objective	Outcome		Hypothesis	Method of analysis
	Name	Type		
Subgroup analysis 1				
To determine subgroup treatment effects in males versus females	PPSP as defined by the WHO and of ≥4/10 severity	Binary	CBT will be associated with a larger reduction in the prevalence of PPSP in females compared to males	Logistic regression
Subgroup analysis 2				
To determine subgroup treatment effects of any open versus no open fracture	PPSP as defined by the WHO and of ≥4/10 severity	Binary	CBT will be associated with a larger reduction in the prevalence of PPSP in participants with open fractures compared to participants with only closed fractures	Logistic regression
Subgroup analysis 3				
To determine subgroup treatment effects of higher vs. lower SPOC scores	PPSP as defined by the WHO and of ≥4/10 severity	Binary	CBT will be associated with a larger reduction in the prevalence of PPSP in participants with higher vs. lower SPOC scores	Logistic regression

PPSP = persistent postsurgical pain; WHO = World Health Organization; CBT = cognitive behavioral therapy; SPOC = Somatic Pre-Occupation and Coping.
